# The IS*Apl1*_2_ Dimer Circular Intermediate Participates in *mcr-1* Transposition

**DOI:** 10.3389/fmicb.2019.00015

**Published:** 2019-01-22

**Authors:** Yu-Zhang He, Xing-Ping Li, Yuan-Yuan Miao, Jun Lin, Ruan-Yang Sun, Xiao-Pei Wang, Ya-Ya Guo, Xiao-Ping Liao, Ya-Hong Liu, Youjun Feng, Jian Sun

**Affiliations:** ^1^National Risk Assessment Laboratory for Antimicrobial Resistance of Animal Original Bacteria, South China Agricultural University, Guangzhou, China; ^2^Guangdong Provincial Key Laboratory of Veterinary Pharmaceutics Development and Safety Evaluation, South China Agricultural University, Guangzhou, China; ^3^Department of Animal Science, The University of Tennessee, Knoxville, Knoxville, TN, United States; ^4^Department of Medical Microbiology and Parasitology, Zhejiang University School of Medicine, Hangzhou, China; ^5^Department of General Intensive Care Unit of the Second Affiliated Hospital, Zhejiang University School of Medicine, Hangzhou, China; ^6^College of Animal Sciences, Zhejiang University, Hangzhou, China

**Keywords:** transposition mechanism, *mcr-1*, circular intermediate, IS*Apl1*, colistin resistance

## Abstract

**Objectives:** The mobile colistin resistance gene *mcr-1* is a serious threat to global human and animal health. The composite transposon Tn*6330* and its circular intermediate were proposed to be involved in the spread of *mcr-1* but their roles remain poorly understood.

**Methods:** To further explore the intermediates during the transposition of Tn*6330*, we engineered *Escherichia coli* strains that carry an intact Tn*6330* transposon or its deletion derivatives. PCR assays were performed to detect IR-IR junctions and possible circular intermediates. We carried out transposition experiments to calculate transposition frequency. The transposition sites were characterized by whole genome sequence and ISMapper-based analyses.

**Results:** The presence of an intact Tn*6330* was demonstrated to be essential for the successful transposition of *mcr-1*, although both Tn*6330* and Tn*6330*-ΔIR could form circular intermediates. The insertion sequence junction structure was observed in all constructed plasmids but the IS*Apl1* dimer was only formed in one construct containing an intact Tn*6330*. The average frequency of *mcr-1* transposition in an *E. coli* strain possessing an intact Tn*6330* was ∼10^-6^ per transformed cell. We identified 27 integration sites for the Tn*6330* transposition event. All the transposition sites were flanked by 2 bp target duplications and preferentially occurred in AT-rich regions.

**Conclusion:** These results indicate that *mcr-1* transposition relies on the presence of an intact Tn*6330*. In addition, formation of the tandem repeat IS*Apl1*_2_ could represent a crucial intermediate. Taken together, the current investigations provide mechanistic insights in the transposition of *mcr-1*.

## Introduction

Polymyxins are cationic antimicrobial cyclic polypeptides that have been reintroduced as a final clinical option for carbapenem-resistant bacteria (Li et al., 2006; Poirel et al., 2017a). The mobilized colistin resistance *mcr-1* gene encodes a phosphoethanolamine (PEA) lipid A transferase that catalyzes PEA addition to the 4′-phosphate of lipid A glucosamine moieties (Gao et al., 2016; Feng, 2018; Wei et al., 2018; Xu et al., 2018). This modification confers bacterial resistance to polymyxin (Liu et al., 2016). Since its discovery, *mcr-1* has been detected in over 50 countries and its reservoirs include humans, animals, and foods and associated environments (Sun et al., 2017b; Shen et al., 2018). The coexistence of MCR-1 and extended-spectrum beta-lactamases (ESBL) or carbapenemases poses a challenge to public health safety and clinical therapies (Sun et al., 2018).

The *mcr-1*-bearing plasmids are diverse although the *mcr-1* gene is often accompanied by a highly active 1,070 bp IS*Apl1* element (Sun et al., 2017a; Wang et al., 2018). IS*Apl1* belongs to the IS*30* family containing a 307-amino-acid-long DDE-type transposase surrounded by imperfect terminal inverted repeat sequences (21/27 nucleotide identity) (Tegetmeyer et al., 2008). In general, the *mcr-1-pap2* cassette lacks a flanking IS*Apl1*, possesses one IS*Apl1* immediately upstream or is flanked by two IS*Apl1* elements. The IS*Apl1*-*mcr-1*-*pap2*-IS*Apl1* transposable cassette was named Tn*6330* (Li et al., 2017).

The *mcr-1* gene was most likely mobilized by IS*Apl1* mediated composite transposon (Tn*6330*) (Snesrud et al., 2016, 2018). To demonstrate the function of the composite transposon Tn*6330*, Poirel et al. (2017b) constructed Tn*6330.2* in which the *mcr-1* gene was inactivated with a *bla*_TEM-1_ insertion, and characterized Tn*6330* participating in the mobilization of *mcr-1* gene. A circular intermediate comprised of IS*Apl1*-*mcr-1*-*pap2* was identified as essential for *mcr-1* mobilization and was generated from the downstream IS*Apl1* (Li et al., 2017). However, a circular intermediate does not necessarily require the complete IS*Apl1*. A circular intermediate originating from a truncated IS*Apl1* immediately downstream of *mcr-1* could also be detected (Zhao et al., 2017). Therefore, the circular intermediate for *mcr-1* mobilization is unclear.

Insertion sequence (IS) dimers can be detected by the presence of an inverted repeat (IR) junction, a full copy IS adjacent to a truncated IS or a circular IS (Olasz et al., 1993; Kiss and Olasz, 1999). For example, this has found experimentally by the detection of IR-IR junctions formed by site specific dimerization in tandem IS*30* elements (Kiss and Olasz, 1999). However, it is not known whether an IS*Apl1* dimer is formed during Tn*6330* transposition. To address this issue, we engineered a collection of plasmids bearing Tn*6330* and its derivatives and demonstrated that transposition of *mcr-1* relied on intact Tn*6330* for efficient integration into the *Escherichia coli* genome. Additionally, we found a tandem IS*Apl1* repeat IS*Apl1*_2_-*mcr-1*-*pap2* that could represent a crucial intermediate during Tn*6330* transposition.

## Materials and Methods

### Strains

**FIGURE 1:**
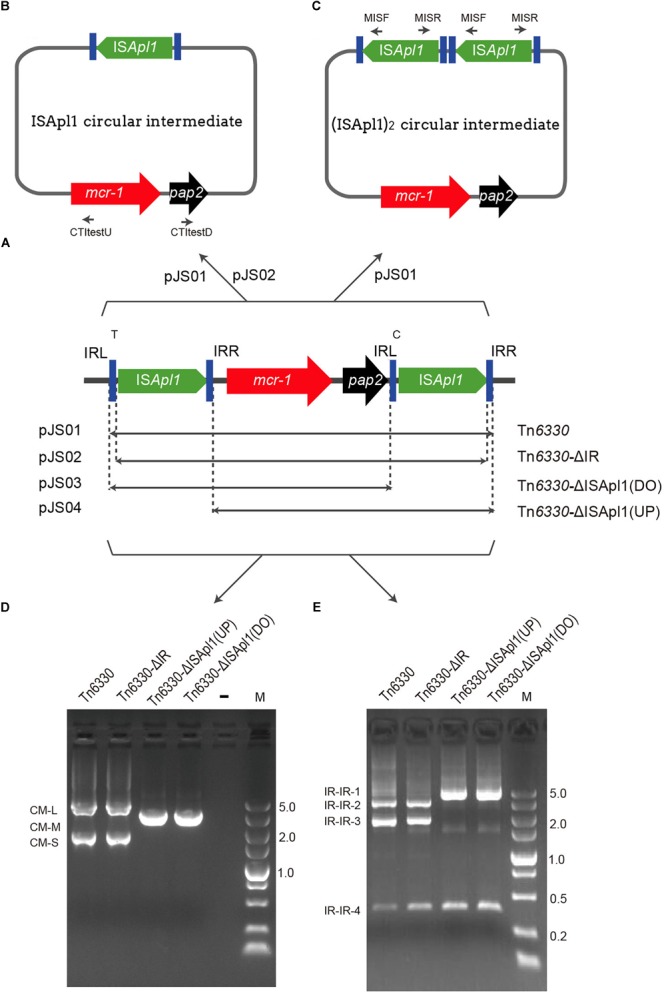
Schematic representation of *mcr*-bearing transposons and verification of the presence of an IS*Apl1-* mediated circular intermediate. **(A)** Structures of Tn*6330* derivatives and plasmid hosts. **(B)** A circular intermediate and **(C)** An IS*Apl1* dimer circular intermediate. **(D,E)** Agarose gel electrophoresis of PCR products generated from screening assays using *Escherichia coli* strains containing the indicated Tn constructs. **(D)** Reverse PCR assay using primers CTI test U and CTI test D to identify IS*Apl1*-*mcr-1*-*pap2* intermediates. CM-L (circular form) represents the remnants of the *pap2*, IS*Apl1* backbone of the suicide plasmid, IS*Apl1* and part of *mcr-1.* **(E)** PCR products generated using primers MISF and MISR to screen for the presence of IR-IR junctions.

*Escherichia coli* MG1655 (wild-type) and *E. coli* MG1655 (*recA*::Km) strains were used as host strains in the transposition experiments (Table 1; Gerdes et al., 2003). *E. coli* strain BW25141 strain contained the *pir* gene possessing an R6K replication origin (Datsenko and Wanner, 2000) and was used as a host to construct suicide plasmids bearing Tn*6330* and derivatives (Table 1). The *E. coli* swine strain CBJ3C was used as a template to amplify Tn6330 (Table 1). The Tn*6330* upstream IS*Apl1* (5′-TTTCCAA-3′) and downstream IS*Apl1* (5′-CTTCCAA-3′) differed by only one bp (underlined) (Figure 1).

**Table 1:** Strains and plasmids used in this study.

Strain	Description	Reference
*E. coli* MG1655 (wild-type)	K-12 strain F^-^ λ^-^ *ilvG rfb-50 rph-1*	Gerdes et al., 2003
*E. coli* MG1655 (*recA*::Km)	K-12 strain F^-^ λ^-^ *ilvG rfb-50 rph-1 recA*	Gerdes et al., 2003
*E. coli* BW25141	F-, *ΔaraDB567, ΔlacZ_4787_*(::*rrnB3*), *ΔphoBR580, λ-, galU95*,Δ*uidA3*::*pir*+, *recA1, endA9*(del-*ins*)::FRT, *rph-1, ΔrhaDB568, hsdR514*	
*E. coli* CBJ3C	Clinical isolate carrying Tn*6330*	
pSV03	Cm^R^, replication origin from *E. coli* plasmid R6K; requires the R6K initiator protein *pir* for replication	This study
pKD4	Lambda red recombinase system template plasmid	
pKD46	Lambda red recombinase system template plasmid	
pJS01	Suicide plasmid (R6K replication origin) contains IS*Apl1*-*mcr-1*-*pap2*-IS*Apl1*	This study
pJS02	Suicide plasmid (R6K replication origin) contains Tn*6330* (IS*Apl1*-*mcr-1*-*pap2*-IS*Apl1*) without upstream IRL and downstream IRR	This study
pJS03	Suicide plasmid (R6K replication origin) contains IS*Apl1*-*mcr-1*-*pap2*	This study
pJS04	Suicide plasmid (R6K replication origin) contains *mcr-1*-*pap2-* IS*Apl1*	This study

### Plasmid Construction

Tn*6330*, Tn*6330*-ΔIR, Tn*6330*-ΔIS*Apl1*(DO) (downstream) and Tn*6330*-ΔIS*Apl1*(UP) (upstream) were cloned into suicide plasmid pSV03 (Rakowski and Filutowicz, 2013) and were named pJS01, pJS02, pJS03, and pJS04, respectively (Table 1, Figure 1A, and Supplementary Figure S1). Primers used for plasmid constructions are listed in Table 2.

**Table 2:** Primers used for plasmid construction.

Primer	Sequence ( 5′ → 3′)^a^	Reference
TUtestF-BglII	TACGCAGATCTACTACTGTGGCTAAGCCTCAAC	This study
TUtestR-XhoI	TACGCCTCGAGACGGAGAGTAACAACACGATGC	This study
R6K-BglII	TACGCAGATCTCCATGTCAGCCGTTAAGTGT	This study
R6K-XhoI	TACGCCTCGAGGTTGATCGGCACGTAAGAGG	This study
R6K-BamHI	TACGCGGATCCGTTGATCGGCACGTAAGAGG	This study
R6K-EcoRI	TACGCGAATTCCCATGTCAGCCGTTAAGTGT	This study
P1	GGCTGCAGACGCACAGCA	This study
IR-F	TTTTTTGAAGTAAACTTCATAAGGTGTTTTCCAACC	This study
CmR-F	ACCTTATGAAGTTTACTTCAAAAAAAGACTAAAAGAGAAGGGAGT	This study
Sac-R	CCAAGCGAGCTCGATATCAA	This study
Sac-F	TTGATATCGAGCTCGCTTGG	This study
CmR-R	ATTATATTCTAGTTGATGAGTACTTCTTTTTCTCTTTAAGTTGAGGCTTAGCC	This study
IR-R	AAAAAGAAGTACTCATCAACTAGAATATAATTTTGTTTCCACAC	This study
P2	ATTGCTGTGCGTCTGCAGCCA	This study
UP-TF	AGACTAAAAGAGAAGGGAGTG	This study
UP-TR	CGATTAAACTTGTTCACCCTTC	This study
DO-F	CTCTCAAGTGTATATTCAGTATGGG	This study
DO-R	CTCTTTAAGTTGAGGCTTAGCC	This study
CTItestU	CGATGATAACAGCGTGGTGATC	This study
CTItestD	TTGCCGATGCTTGATAGTATGC	This study
MIS-F	CAATCAGTGGAGCGAAGTTG	This study
MIS-R	CTGTTTTGTGCGTTCGTTGC	This study

*^*a*^Restriction sites are underlined.*

In pJS01, the structure of IS*Apl1*-*mcr-1*-*pap2*-IS*Apl1* and flanking sequences were amplified by PCR using primers TUtestF-BglII and TUtestR-XhoI with *E. coli* CBJ3C template DNA. Primers R6K-BglII and R6K-XhoI were used to amplify the backbone of pSV03, which includes the conditional replication origin R6K and chloramphenicol resistance gene (CmR). A ligation was performed giving rise to recombinant plasmid pJS01. The plasmid was transformed into *E. coli* BW25141 and selected on Luria-Bertani (LB) agar plates supplemented with 25 μg/ml chloramphenicol (Cm). The integrity of both IS*Apl1* elements and *mcr-1* was confirmed by PCR and sequence analysis.

The plasmid pJS02 was used to amplify a partial *mcr-1, pap2*, and IS*Apl1*-ΔIRR (IR right) fragment using primers P1 and IR-F. It was constructed using a fragment containing Cm^R^ that was amplified using primers CmR-F that lacked the 27 bp IRR and SacR containing a *SacI* site. The amplicons were connected by overlapping PCR resulting in a DNA fragment of the downstream IS*Apl1* lacking the 27 bp IRR that was bounded by *PstI* and *SacI* restriction enzyme sites. The sequence containing a fragment of the upstream IS*Apl1* without a 27bp IRL (IR left) containing *PstI* and *SacI* restriction sites at the ends was obtained in the same manner using primers Sac-F, CmR-R, IR-R and P2. The amplified fragments were digested with *PstI* and *SacI* and joined using T4 ligase. Plasmid pJS02 was confirmed as described above for pJS01.

Plasmids pJS03 and pJS04 were derived using primers UP-TR, UP-TF and DO-F and DO-R to amplify DNA fragments lacking the downstream copy of IS*Apl1* or upstream copy of IS*Apl1*, respectively. After self-ligation, the plasmids were screened and confirmed as for pJS01 above.

### The Detection of Circular Intermediate and IR-IR Junction

All constructed plasmids carrying Tn*6330* or its derivatives were tested for the ability of IS*Apl1-mcr-1* to generate circular forms using reverse PCR with primers CTItestU and CTItestD that targeted *mcr-1* and *pap2*, respectively. To identify IR-IR junctions, PCR and Sanger sequencing were performed using primers MIS-F and MIS-R which were directed outward from IS*Apl1* (Table 2).

### Transposition Assays

Transposition assays were performed as previously described (Bontron et al., 2016). In brief, suicide plasmids pJS01, pJS02, pJS03, and pJS04 were electroporated into *E. coli* MG1655 (wild-type) and *E. coli* MG1655 (*recA*::Km) using a Biorad MicroPulser (Hercules, CA, United States) and the protocol supplied by the manufacturer. The bacteria were suspended in 1ml LB and incubated for 1 h at 37°C with agitation and serially diluted onto LB-agar containing 2 μg/ml colistin to select for transposition events. The presence of the full-length transposon Tn*6330* was confirmed using PCR with primers in Supplementary Table S1. The transposition frequencies were calculated by dividing the number of transposition events by the number of transformed cells in triplicate (Milewska et al., 2015).

### Mapping of Transposon Insertion Sites

**FIGURE 2:**
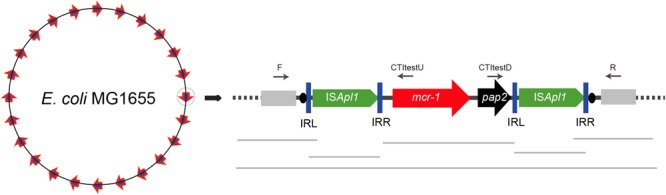
Schematic for determination of the transposition site by WGS and PCR. Sequences of *E. coli* MG1655 are shown as rectangles in light gray. The inverted repeats (IRL and IRR) are represented as blue vertical bars and DRs as black ovals.

Transposon insertion sites in *E. coli* MG1655 (*recA*::Km) were identified from random genomic DNA samples of each confirmed transposant prepared from overnight cultures using the TIANamp Bacteria DNA Kit (Tiangen, Dalian, China). The DNA of all the transposants was then mixed together into a single pool and a 300-bp library was constructed for Illumina paired-end sequencing (Illumina, San Diego, CA, United States). Illumina sequences were assembled *de novo* using SOAP software (Luo et al., 2012). The contigs carrying *mcr-1* and IS*Apl1* fragments were concatenated through the ISmapper analysis (Hawkey et al., 2015). Then the gaps were closed using PCR mapping and Sanger sequencing as shown in Figure 2. The primers targeted in the sequences of chromosome and *mcr-1-pap2* was designed in different insert regions (Supplementary Table S1 and Table 1) to determine transposition sites.

To characterize the genetic context of Tn*6330* in clinical strains, the sequences carrying Tn*6330* in GenBank were collected. For each transposition event, the relative frequencies of each A and T, and G and C of the region extending from 50 nucleotides upstream to 50 nucleotides downstream from the insert target were calculated and plotted on a line graph (Tang et al., 2017). The pictures of the relative frequencies of the bases at each position were generated with the Pictogram program^1^.

## Results

### Transposition of the Composite Transposon

We identified the transposition abilities of the Tn*6330* derivatives by cloning into suicide plasmids that were then electroporated into strain BW25141 (*pir*^+^). These suicide plasmids were transformed into two *E. coli* recipient strains MG1655 (wild-type) and MG1655 *(recA*::Km). Survival was contingent upon transposition of the selectable markers into the host genome. The transposition frequencies of pJS01 into both *E. coli* strains occurred at 2.7 × 10^-6^ per transformed cell. PCR and Sanger sequencing results showed that the downstream (5′-CTTCCAA-3′) and upstream (5′-TTTCCAA-3′) of IS*Apl1* in Tn*6330* in the insertion sites were different, indicating complex transposition events (data not shown). In contrast, all other constructs failed to generate cell survival in the presence of colistin (2 μg/ml). This indicated that Tn*6330*-ΔIR, Tn*6330*-ΔIS*Apl1*(DO), and Tn*6330*-ΔIS*Apl1*(up) could not transpose successfully.

Interestingly, we found evidence for the formation of circular intermediates containing the IS*Apl1-mcr-1-pap2* structure (CM-S) from plasmids harboring Tn*6330* and Tn*6330*-ΔIR. However, if the upstream or downstream IS*Apl1* was removed, no circular form could be detected (Figures 1B,D and Table 3).

**Table 3:** Transposition frequencies of suicide plasmids bearing *mcr-1* genes.

Plasmid	Transposase	Reverse PCR	IR-IR junction	Transposition frequency (wild type)^a^	Transposition frequency (recA::Km)
pJS01	+	+	+	2.78 × 10^-6^	2.71 × 10^-6^
pJS02	–	+	+	–	–
pJS03	+	–	+	–	–
pJS04	+	–	+	–	–

**FIGURE 3:**
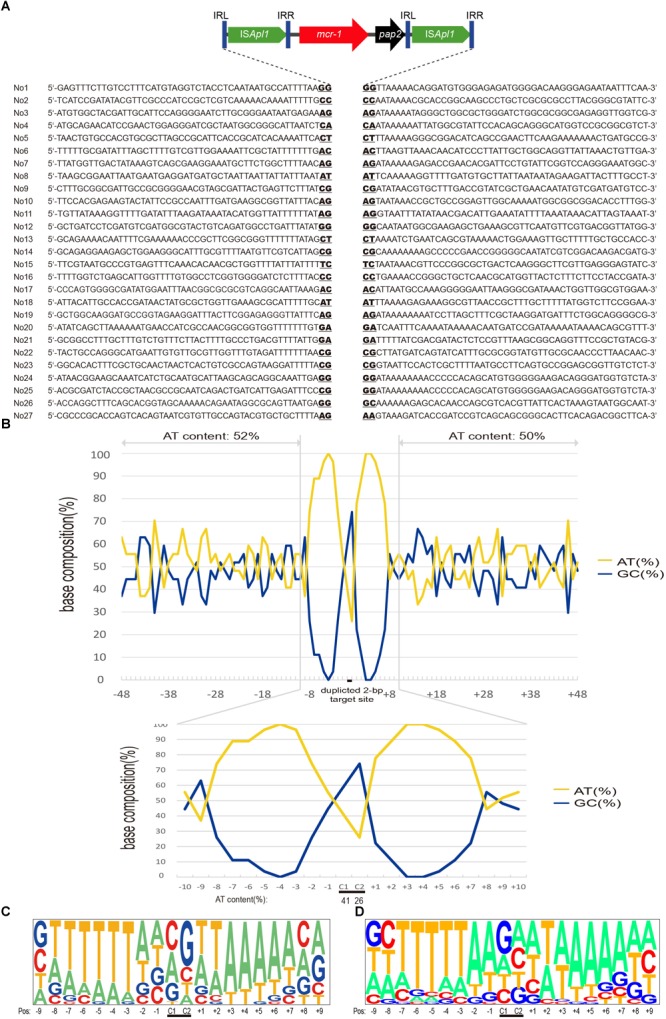
Target site analyses of Tn*6330* transposons. **(A)** Molecular characterization of 27 transposition events of Tn*6330* transposons in *E. coli* MG1655 (*recA*::Km). The duplicated 2-bp target site is underlined in the context of the surrounding 48 nucleotides upstream and downstream of the target sites. **(B)** Statistical*( analyses of the 27 transposition sites. The percentage of AT and GC at each position from 48 nucleotides upstream to 48 nucleotides downstream of the target site are shown. The 2-bp duplicated target site (c1 and c2) are indicated by black bars. The AT and GC percentages of regions spanning positions –48 to –3 and positions +3 to +48 and that of the region spanning positions –2 to 2 are indicated in the upper and lower graphs, respectively. Relative nucleotide frequencies at each target site deduced from the **(C)** 27 experimental transposition events shown in **(A)** and **(D)** from 26 Tn*6330* transposons in clinical isolates obtained from GenBank (Supplementary Table S2).)*

All these transposition events generated IR-IR junctions were separated by 2 bp spacers (Figures 1C,E, 3). This would be possible through the formation of IS*Apl1* dimers (pJS01, Figure 1C), a truncated IS*Apl1* next to a truncated IS*Apl1* (pJS02) or a circularized IS*Apl1* that was possible with all constructs (Kiss and Olasz, 1999).

### Target Site Specificity

Whole genome sequence (WGS) and ISMapper-based analyses revealed 27 integration sites. The Illumina reads have been deposited in GenBank under accession no. SRR8365224. The insert locations of the *mcr-1* gene were further confirmed by PCR and Sanger sequencing. The majority of these events (24/27) generated 2-bp duplications and occurred in AT-rich regions with a high preference for insertion between T and A. The mean AT content extending in each direction from the 2-bp target sites (–50 to –2 bp and +2 to +50 bp) were 52 and 50%, respectively (Figure 3). In addition, the AT content increased nearer the target site and was 100% at positions –4, +3 and +4 and 74 to 96% at positions –7, –6, –5, –2, +1, +2, +5, +6 and +7. At the duplicated target site positions (c1 and c2) the AT content was lower (26 to 41%) (Figure 3B).

### Distribution of Tn*6330*-Like Transposons in Enterobacteriaceae

To further characterize the transposon events in clinical strains, we collected sequences harboring the Tn*6330*-like structures from GenBank in isolates from more than ten regions including China, Hong Kong, Taiwan, Japan, Malaysia, Thailand, United States, Italy, Germany, Switzerland, Argentina, and Canada (Supplementary Table S2). We found that 47 sequences had 2-bp target directed repeats, a characteristic signature of transposition events of Tn*6330*-like transposons. The AT preferences of Tn*6330* insertions were similar to that *in vitro* mobilization assays presented above (Figure 3D).

## Discussion

In this study we demonstrated the functionality of Tn*6330* transposition from plasmids where cell survival was dependent on transposition of the *mcr-1* selective marker. The intact Tn*6330* in plasmid pJS01 transposed efficiently into the *E. coli* chromosome. Transposition occurs *via* a highly reactive intermediate such as IS*30*_2_ and provides a molecular model for IS*30*-like transposition. This also relied on a circular intermediate carrying an active IR-IR junction (Olasz et al., 1993; Kiss and Olasz, 1999). The IS*Apl1* element in Tn*6330* belongs to the IS*30* family so we examined the role of IS*Apl1*_2_ carrying joined IRs in IS*Apl1-* mediated transposon. Previous studies provided evidence that the reverse PCR amplicon IS*Apl1*-*mcr-1*-*pap2* acted as a circular intermediate (Li et al., 2017; Zhao et al., 2017). However, this could not distinguish between that structure and (IS*Apl1*)_2_-*mcr-1*-*pap2*. All four of our plasmid constructs generated IR-IR junctions.

The genuine IS*30*-like circular intermediate of Tn*6330* composed of (IS*Apl1*)_2_-*mcr-1*-*pap2* was only formed from pJS01 (Figure 1C). This was dependent upon the IS*Apl1* IR-IR junction and the production of the transposase for successful transposition into the *E. coli* chromosome (Kiss and Olasz, 1999). Plasmids pJS02, pJS03 and pJS04 could not form the IS*Apl1*_2_-*mcr-1*-*pap2* circular intermediates and failed to transpose. This would also explain that *mcr-1* in the absence of flanked copies of IS*Apl1* or just one copy of IS*Apl1* originated from an ancestral Tn*6330* (Snesrud et al., 2018). The transposition of *mcr-1* relied on an intact Tn*6330*.

Transposition frequencies of suicide plasmids carrying Tn*6330* were high at rates of 10^-6^ per transformed cell both in wild type and *recA* mutant MG1655 strain. The relatively high Tn*6330* transposition frequency together with frequent insertion into transmissible plasmid targets might explain why the *mcr-1* gene is globally prevalent. Tn*6330* has been found in *E. coli, Salmonella enterica, Klebsiella pneumoniae, Citrobacter freundii*, and *Citrobacter braakii* (Supplementary Table S2). The composite transposon Tn*6330* might lose one or both copies of IS*Apl1* through illegitimate recombination giving rise to different types of genetic contexts such as IS*Apl1*-*mcr-1*-*pap2, mcr-1*-*pap2*, ΔTn*6330* and others (Snesrud et al., 2016). Loss of IS*Apl1* seems to be conducive to *mcr-1* maintenance increasing the stability of this gene in the host genome or plasmids and raising the risk of *mcr-1* dissemination.

The target site of Tn*6330* was AT rich in the 6 bp surrounding the duplicated target site. In *E. coli* clinical isolates, the same features were present both in plasmids and chromosomal regions consistent with previous works (Snesrud et al., 2016, 2018; Poirel et al., 2017b). Both the experimental transposants and *E. coli* clinical isolates showed a high frequency of T on the upstream and A on the downstream sides of the Tn*6330* target site. These findings contrast with previous descriptions that indicated target site duplication always carried a C or a G or both suggesting a relatively even distribution of A, T, G and C.

Though Poirel et al. (2017b) have demonstrated the mobility of the *mcr-1* gene by transposition, some differences exist in our study: (1) we found the suicide plasmids harboring *mcr-1* could successfully transpose into the bacterial chromosome using the colistin resistant phenotype during the process of transposition. We found no visible toxic effects to the presence of MCR-1. Toxic effects of MCR-1 that limited colonization of *mcr-1* in regular bacterial cells might be caused by high plasmid copy number (Yang et al., 2017). (2) We characterized 27 transposon sites using WGS and ISMapper. Compared with previous digestion and inverse PCR strategies, the ISMapper method might be more convenient and efficient (Poirel et al., 2017b). (3) The regions of the downstream IS*Apl1* (CTTCCAA) were different from the upstream IS*Apl1* (TTTCCAA) in all the transposants; the same as initial Tn*6330* in pJS01. This result suggested that the transposition events were not from the IS*Apl1*-*mcr-1*-*pap2* circular form. This was further evidence for an IS*Apl1* dimer-mediated composite transposon (Snesrud et al., 2018). In addition, our study for the first time indicates that an IS*Apl1* dimer plays a crucial role as a genuine circular intermediate. This contrasts with previous studies indicating that the IS*Apl1*-*mcr-1*-*pap2* circular form results in the transposition of *mcr-1* (Li et al., 2017; Zhao et al., 2017). A reverse PCR amplicon does not completely characterize a circular intermediate since it cannot identify the IS-IS junction.

In summary, our results further verified that the transposition of *mcr-1* is only mediated by an intact Tn*6330* but not the amplicon identified by reverse PCR, the IS*Apl1*-*mcr-1*-*pap2* circular form. In addition, the IS*Apl1* dimer IS*Apl1*_2_-*mcr-1*-*pap2* represents a crucial intermediate in *mcr-1* transmission. Future studies will focus on the regulatory mechanisms of Tn*6330* transposition in the search for a viable path to block the spread of the colistin resistance gene *mcr-1*.

## Author Contributions

JS designed this project. Y-ZH, Y-YM, X-PW, and Y-YG performed the experiments. Y-ZH, X-PLi, YF, and JS analyzed the data. X-PLi and R-YS made the figures. X-PLi wrote this manuscript. JL, X-PLiao, YF, and JS edited and revised the manuscript. Y-HL coordinated the whole project.

## Conflict of Interest Statement

The authors declare that the research was conducted in the absence of any commercial or financial relationships that could be construed as a potential conflict of interest. The reviewer BZ declared a shared affiliation, with no collaboration, with one of the authors, YF to the handling Editor at the time of review.

## Footnotes

1 http://genes.mit.edu/pictogram.html
