# Supplementary material for: The ISApl12 Dimer Circular Intermediate Participates in mcr-1 Transposition
Source: Front Microbiol. 2019 Jan 22;10:15. doi: 10.3389/fmicb.2019.00015 (PMC6349718; doi:10.3389/fmicb.2019.00015)
Supplement: Supplementary file 1 [file Data_Sheet_1.docx]

**Supplementary Information**

**
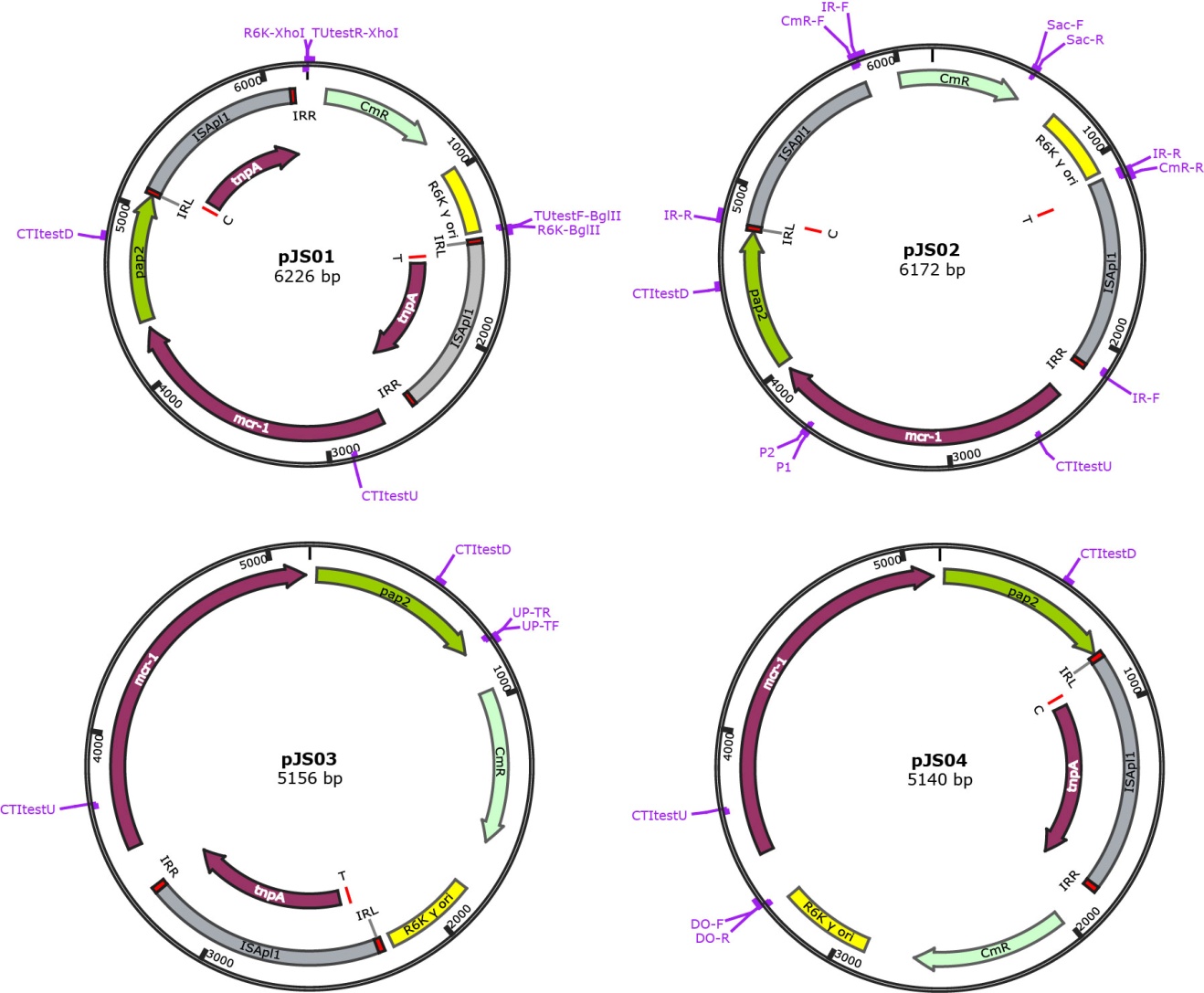
**

**Figure S1**. Structures of constructed plasmids of pJS01, pJS02, pJS03 and pJS04 carrying *mcr*-*1* gene.

**Table S1.** Target site test primers used in this study.

| Target site | primers | Sequence (5'→3') |
| --- | --- | --- |
| NO1 | NO1F  NO1R | GGCGGTCTATAAACGCACCA  GGACGACCGATTGATTCCCA |
| NO2 | NO2F  NO2R | ATCGTCTGGAACAGCGTCTG  TCGGAGGCGTTAGAGATAAGC |
| NO3 | NO3F  NO3R | ACGGTATTGACGATCCTCACTT  GCTGTTCTTCTGGCGGTAAC |
| NO4 | NO4F  NO4R | GGTGGCAACATAGGCTATACTC  GCAAAGCGACGGGAACAATT |
| NO5 | NO5F  NO5R | AAGTGAGCAGCAGCGATTAG  TGTTGCCAGCGAGTATTCATAA |
| NO6 | NO6F  NO6R | CATCACGACCGAGGAAGACT  TATAGCCGAGACCACCGAAG |
| NO7 | NO7F  NO7R | CGACATTCTACCGCCTCTGA  TTGCTGGTGGACGAAAGATTG |
| NO8 | NO8F  NO8R | TTCATCACTGCTGGAGATTACC  GACAAGTGAAAACATGCACCC |
| NO9 | NO9F  NO9R | GCCAGATATGTCGCCATTGAT  CCCATTTCGGTGATTCAGATTG |
| NO10 | NO10F  NO10R | AGGGTCATTGCGAATTATCTGA  CGGACCACGGATGTCATAAC |
| NO11 | NO11F  NO11R | GACAACATTCGTGCCATCAGT  GCGTCATTGCCTCTTCACTC |
| NO12 | NO12F  NO12R | TACTACGGAACTTCGCTGAGT  CCGATGCTATTGCTGAACCTT |
| NO13 | NO13F  NO13R | GCATCCTGACGACAATCTACG  GCGACCAGAGAAACCAACATAA |
| NO14 | NO14F  NO14R | CGTGGATGTTCGTCAGTTAAGT  GAGATCATCGCCGACATAAGC |
| NO15 | NO15F  NO15R | AATGCGGACCTGATGGCTAT  CTGGCTTCGTTACTCGTGAC |
| NO16 | NO16F  NO16R | GTAAGTGGTGTGGCGTCTTC  ATGCGTTGACCGATAGTTAAGT |
| NO17 | NO17F  NO17R | CCTTCCATCTGGCGAATCATAA  AGGCGTGGCGATAATATAATCA |
| NO18 | NO18F  NO18R | ATGACGCAGGTCGTGAGTTA  CCGCAACTGATTGAAGGCTATA |
| NO19 | NO19F  NO19R | GGCTTCACCGTCTCAAGAATG  GCAACACAACATCACGAATGG |
| NO20 | NO20F  NO20R | CAGGCGTCACAATCGGAATG  AGCGGGTCAGTTCTTCGTTAT |
| NO21 | NO21F  NO21R | TTCCAGCGGTGAAGTTACATC  GATGAACTCGACGGTAGTCG |
| NO22 | NO22F  NO22R | CGTTCCGCCACCCTAATTAG  ATATGAATGGCTGGCAAGGATG |
| NO23 | NO23F  NO23R | ACTTCGGCTCCGTTGATAACT  TTCCATCACCTGTCGGCATAT |
| NO24 | NO24F  NO24R | CGTTATCGGCATGGTTAAGGAT  TGTGAATGAACGGTAACGCAAT |
| NO25 | NO25F  NO25R | TCCTGCCATAAACTCGCGTT  CCAGCGTGGAGATCACAATC |
| NO26 | NO26F  NO26R | TGTCGGTGGTAGTGGCAGAA  CGCTTCAGTAAGGTCACAATGC |
| NO27 | NO27F  NO27R | CGCCGTTCATCATAACATCAGA  TTAGCGAACTGGAGACAATGC |

**Table S2.** Plasmids and genome sequences bearing two or more copies of IS*Apl1*, searched in Genbank database.

| Plasmids/  Strains | Size (bp) | Location | Specials | Source | Region | IS*Apl1*  (up) | *pap2* | IS*Apl1*  (down) | DR | *mcr*-  type | Acc. No. |
| --- | --- | --- | --- | --- | --- | --- | --- | --- | --- | --- | --- |
| pNG14043 | 42,941 | X4 | *Salmonella enterica* | - | Taiwan | 1 | 1 | 1 | AG |  | [KY120363](https://www.ncbi.nlm.nih.gov/nucleotide/1135520350?report=genbank&log$=nuclalign&blast_rank=73&RID=PDNP126U01R) |
| pRC960-2 | 65,538 | I2 | *Shigella flexneri* | - | China | 1 | 1 | 1 | GA |  | [KY784668](https://www.ncbi.nlm.nih.gov/nucleotide/1204566068?report=genbank&log$=nuclalign&blast_rank=3&RID=NWDGHMGN015) |
| pSh487-m4 | 63,512 | I2 | *Shigella sonnei* | - | China | 1 | 0 | 1 | GA |  | KY363996 |
| pMCR-M19241 | 61,584 | I2 | *E. coli* | - | Argentina and Canada | 1 | 1 | 1 | GA | *mcr-*1.5 | KY471311 |
| pMCR-M17059 | 61,531 | I2 | *E. coli* | - | Argentina and Canada | 1 | 1 | 1 | GA | *mcr-*1.5 | [KY471310](https://www.ncbi.nlm.nih.gov/nucleotide/1202278504?report=genbank&log$=nuclalign&blast_rank=2&RID=R0S595AP016) |
| pMCR-M15049 | 61,198 | I2 | *E. coli* | - | Argentina and Canada | 1 | 1 | 1 | GA | *mcr-*1.5 | [KY471308](https://www.ncbi.nlm.nih.gov/nucleotide/1202278330?report=genbank&log$=nuclalign&blast_rank=3&RID=R0S595AP016) |
| pMTY17668-MCR1.5 | 62,375 | I2 | *E. coli* | Infant | Janpan | 1 | 1 | 1 | GA | *mcr-*1.5 | [AP018110](https://www.ncbi.nlm.nih.gov/nucleotide/1194641053?report=genbank&log$=nuclalign&blast_rank=4&RID=R0S595AP016) |
| pECJS-59-244 | 243,572 | HI2 | *E. coli* | - | China | 1 | 1 | 1 | AG/TC |  | KX084394 |
| pS38/ CTX-M-1 | 247,885 | HI2 | *E. coli* | Poultry meat | Italy | 1 | 1 | like | AA |  | KX129782 |
| p14408_M1 | 238,073 | HI2 | *E. coli* | - | Germany | 1 | 0 | 1 | AG/CG |  | [LT599829](https://www.ncbi.nlm.nih.gov/nucleotide/1129837533?report=genbank&log$=nuclalign&blast_rank=78&RID=PDNP126U01R) |
| p19M12 | 232,345 | HI2 | *E. coli* | - | Switzerland | 1 | 0 | reversed | TT/CG |  | KY689632 |
| p100R | 256,260 | HI2 | *E. coli* | - | Switzerland | 1 | 0 | 1 | AT |  | KY689633 |
| pEC2-4 | 235,403 | HI1 | *E. coli* | - |  | 1 | 1 | 1 | CT/TG |  | CP016184 |
| pMCR_1511 | 57,278 | IncP | *Klebsiella pneumoniae* | Hospital sewage | China | 1 | 1 | 1 | AC |  | [KX377410](https://www.ncbi.nlm.nih.gov/nucleotide/1098552673?report=genbank&log$=nuclalign&blast_rank=89&RID=PDNP126U01R) |
| pHYEC7-mcr1 | 97,559 | Phage-like | *E. coli* | - | China | 1 | 1 | 1 | TC |  | [KX518745](https://www.ncbi.nlm.nih.gov/nucleotide/1118591010?report=genbank&log$=nuclalign&blast_rank=79&RID=PDNP126U01R) |
| pMCR-1-P3 | 97,386 | Phage-like IncY | *E. coli* | - | China | 1 | 1 | reversed | AT |  | [KX880944](https://www.ncbi.nlm.nih.gov/nucleotide/1118589944?report=genbank&log$=nuclalign&blast_rank=80&RID=PDNP126U01R) |
| pSCC4 | 44,155 | Unknown | *Citrobacter braakii* | Chicken | China | 1 | 1 | 1 | GG/CG |  | CP021078 |
| EC590 | 4,617,703 | Chromosome (3 copy) | *E. coli* | - | Malaysia | 1 | 1 | 1 | TC |  | CP016182 |
| ECCTRSRTH06 | 110,597 | - | *E. coli* | Homo sapiens | Thailand | 1 | 1 | 1 | AA |  | NQCP01000078.1 |
| pEGY1-MCR-1 | 228,947 | HI2 | *E. coli* | Food | USA | 1 | 1 | 1 | TG/CG |  | CP023143.1 |
| SZH29-1 | 32,630 | - | *E. coli* | - | Hong Kong | 1 | 1 | 1 | CC |  | NIFV01000761.1 |
| SZM334-1 | 42,826 | - | *E. coli* | - | Hong Kong | 1 | 1 | 1 | CC |  | NIFX01000135.1 |
| SZM457-1 | 41,244 | - | *E. coli* | - | Hong Kong | 1 | 1 | 1 | CT |  | NIFU01000125.1 |
| SZM584-1 | 14,1251 | - | *E. coli* | - | Hong Kong | 1 | 1 | 1 | TC |  | NIFT01000160.1 |
| BJ10 | 12,323 | - | *E. coli* | patient | China | 1 | 1 | 1 | TG |  | LWQZ01000105.1 |
| SZM531-1 | 68,216 | - | *E. coli* | - | Hong Kong | 1 | 1 | 1 | GC |  | NIFR01000112.1 |
| pD36-1 | 188,310 | HI2 | *Citrobacter* |  | China | 1 | 1 | 1 | AA |  | MF083142 |
| pEHS30-1 | 179,444 | - | *E. coli* |  | Hong Kong | 1 | 1 | 1 | AC |  | KX772391 |
| pHNGDF36-1 | 52,740 | - | *E. coli* |  | China | 1 | 1 | 1 | GT |  | MF978389 |
| pHNSHP41 | 156,569 | HI2 | *E. coli* |  | China | 1 | 1 | 1 | TC |  | MF784460 |
| pMCR1_WCHEC-LL123 | 223,698 | HI2 | *E. coli* |  | China | 1 | 1 | 1 | CT |  | MF678350 |
| 16RP | 6,693 | - | *Raoultella planticola* |  | China | 1 | 1 | 1 | AG/TC |  | MF593954 |
| pWW012 | 151,609 | HI2 | *Salmonella* |  | China | 1 | 1 | 1 | CT |  | CP022169 |
| pC214 | 42,941 | X4 | *Salmonella* |  | China | 1 | 1 | 1 | AG |  | KY120363 |
| unnamed1 | 15,998 | - | *E. coli* |  | China | 1 | 1 | 1 | GT |  | KX528699 |
| p6383 | 61,198 | I2 | *E. coli* |  | Argentina | 1 | 1 | 1 | GA |  | MG594798.1 |
| p1670 | 55,549 | I2 | *E. coli* |  | Argentina | 1 | 1 | 1 | GA |  | MG598814.1 |
